# Comprehensive macroscopic living anatomy of the swine heart: comparative visual approach with virtual dissection

**DOI:** 10.1038/s41598-026-51948-3

**Published:** 2026-05-19

**Authors:** Kimberly P. Rios, Antony M. Moussa, Hayley A. Rios, Mark Rimmer, Yuichiro Miyazaki, Daisetsu Aoyama, Rami N. Aladham, Lily G. Defelice, Peter Hanna, Justin H. Hayase, Wei-Hsin Chung, Shili Xu, Olujimi A. Ajijola, Kalyanam Shivkumar, Shumpei Mori

**Affiliations:** 1 David Geffen School of Medicine, University of California Los Angeles (UCLA), Los Angeles, CA USA; 2 Division of Cardiology, Department of Internal Medicine, Taichung municipal geriatric rehabilitation general hospital, Taichung, Taiwan; 3 Division of Cardiovascular Medicine, Department of Internal Medicine, China Medical University and Hospital, Taichung, Taiwan; 4 Department of Molecular and Medical Pharmacology, David Geffen School of Medicine at UCLA, Los Angeles, CA USA; 5 Crump Institute for Molecular Imaging, David Geffen School of Medicine at UCLA, Los Angeles, CA USA; 6 Jonsson Comprehensive Cancer Center, David Geffen School of Medicine at UCLA, Los Angeles, CA USA; 7 Division of Cardiology, Department of Medicine, David Geffen School of Medicine at UCLA Center for Health Sciences, Suite 43-244, 650 Charles E. Young Dr. South, Los Angeles, CA 90095 USA

**Keywords:** Comparative anatomy, Computed tomography, Heart, Swine, Virtual dissection, Anatomy, Cardiology, Medical research, Physiology

## Abstract

Swine is one of the major large animal species used in translational research. Extensive knowledge has been accumulated on the normal anatomy of the swine heart, highlighting its similarities with the human heart, including its size and coronary arterial system. However, when these hearts are compared three-dimensionally as they lie on each thorax without distortion, remarkable differences are recognized beyond already known similarities. Those differences include, but are not limited to, the cardiac axis, aortic axis, aortic arch plane, aortic arch/pulmonary trunk relationship, atrial appendages extent, pulmonary arteries/veins/trachea/bronchi relationship, left azygos vein drainage, coronary arterial orifices relationship, fossa ovalis/aortic root relationship, and ventricular trabeculations features. In addition to conventional and rough recognition of similarities between the swine and human hearts, detailed knowledge of differences in the living anatomy of the swine and human hearts is especially fundamental for conducting effective in vivo experiments, including fluoroscopic procedures and survival studies with surgical intervention. Also, this knowledge is useful for discussing the feasibility and limitations of applying the insights obtained from the swine heart study to the human heart. Thus, we herein demonstrate comprehensive macroscopic living anatomy of the swine heart compared with that of the human heart using cardiac computed tomographic datasets obtained from live swine, as well as datasets from recovered swine hearts after pressure-perfusion and fixation to maintain their physiological, non-distorted anatomy.

**Supplementary Information:**

The online version contains supplementary material available at 10.1038/s41598-026-51948-3.

## Introduction

**Fig. 1:**
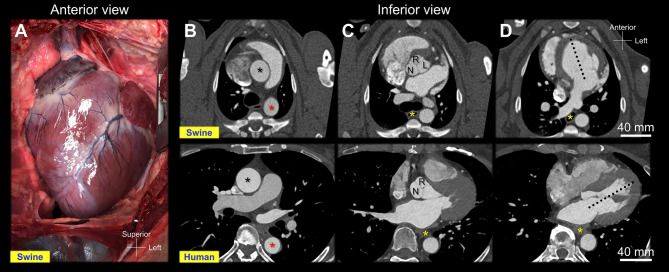
The live swine heart and comparative computed tomographic axial images.The heart of a Yucatan minipig in the supine position is viewed from the anterior direction during the open-chest surgery (**A**). Axial scans obtained from cardiac computed tomography (B-D) of the living swine (upper panels) and human (bottom panels) at the level of the ascending aorta (**B**), aortic root (**C**), and mitral valve (**D**) enhance the differences between species, including the plane involving the ascending (black asterisks) and descending (red asterisks) aorta, location of the esophagus (yellow asterisks), orientation and tilting of the aortic root, and axis of the left ventricle (black dotted lines). L, left coronary aortic sinus; N, non-coronary aortic leaflet; R, right coronary aortic leaflet.

**Fig. 2:**
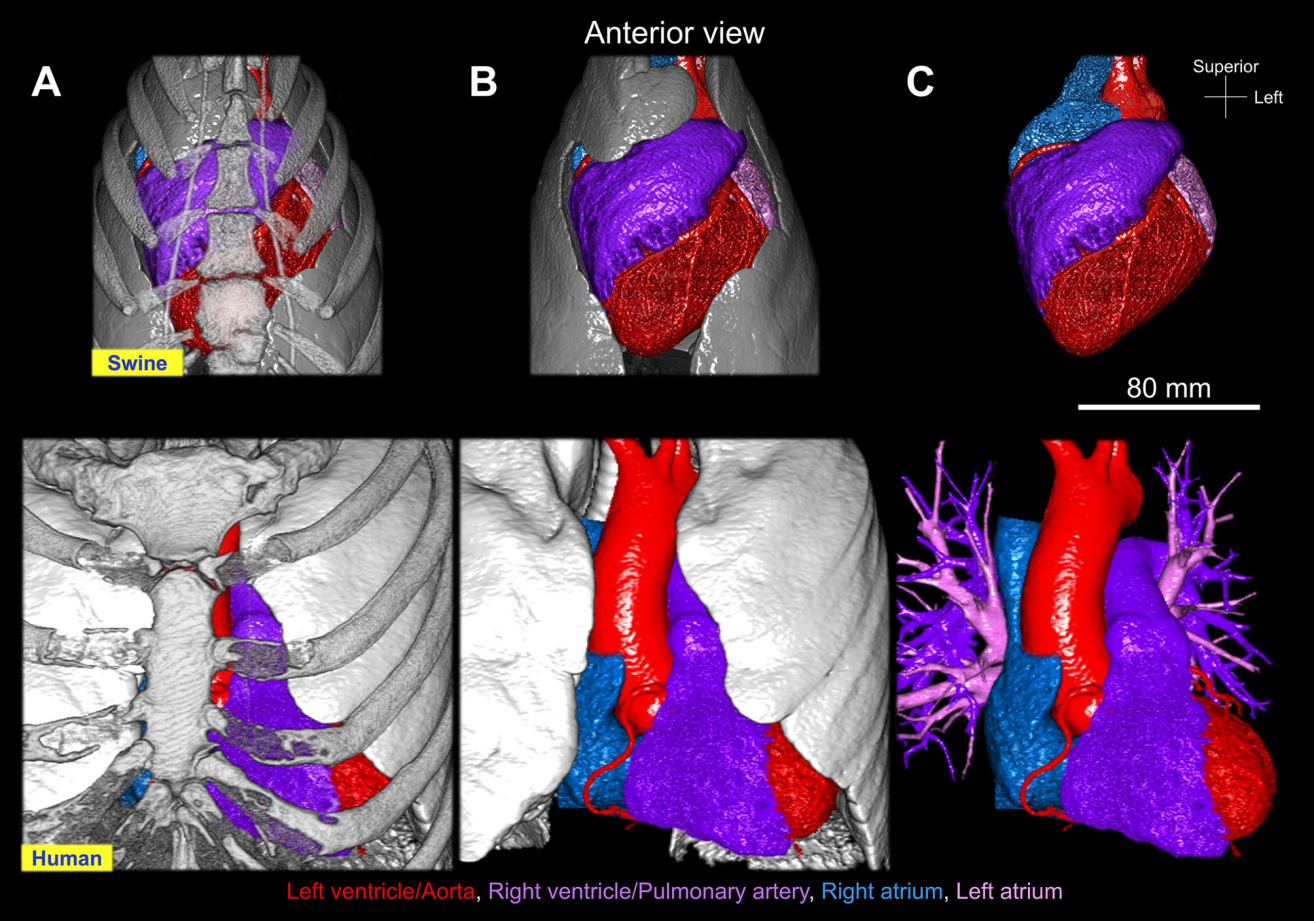
Comparative progressive virtual dissection of the swine and human hearts viewed from the frontal direction. Volume-rendered images of the swine (upper panels) and human (bottom panels) hearts viewed from the anterior direction (**A**-**C**) are demonstrated with progressive virtual dissection with the thoracic cage (A), after removal of the thoracic cage (G), and after recovery of the heart (C) to visually denote their similarities and differences in orientation and rotation. Purple, red, blue, and pink structures indicate the right ventricle and pulmonary artery, left ventricle and proximal aorta, right atrium, and left atrium, respectively.

**Fig. 3:**
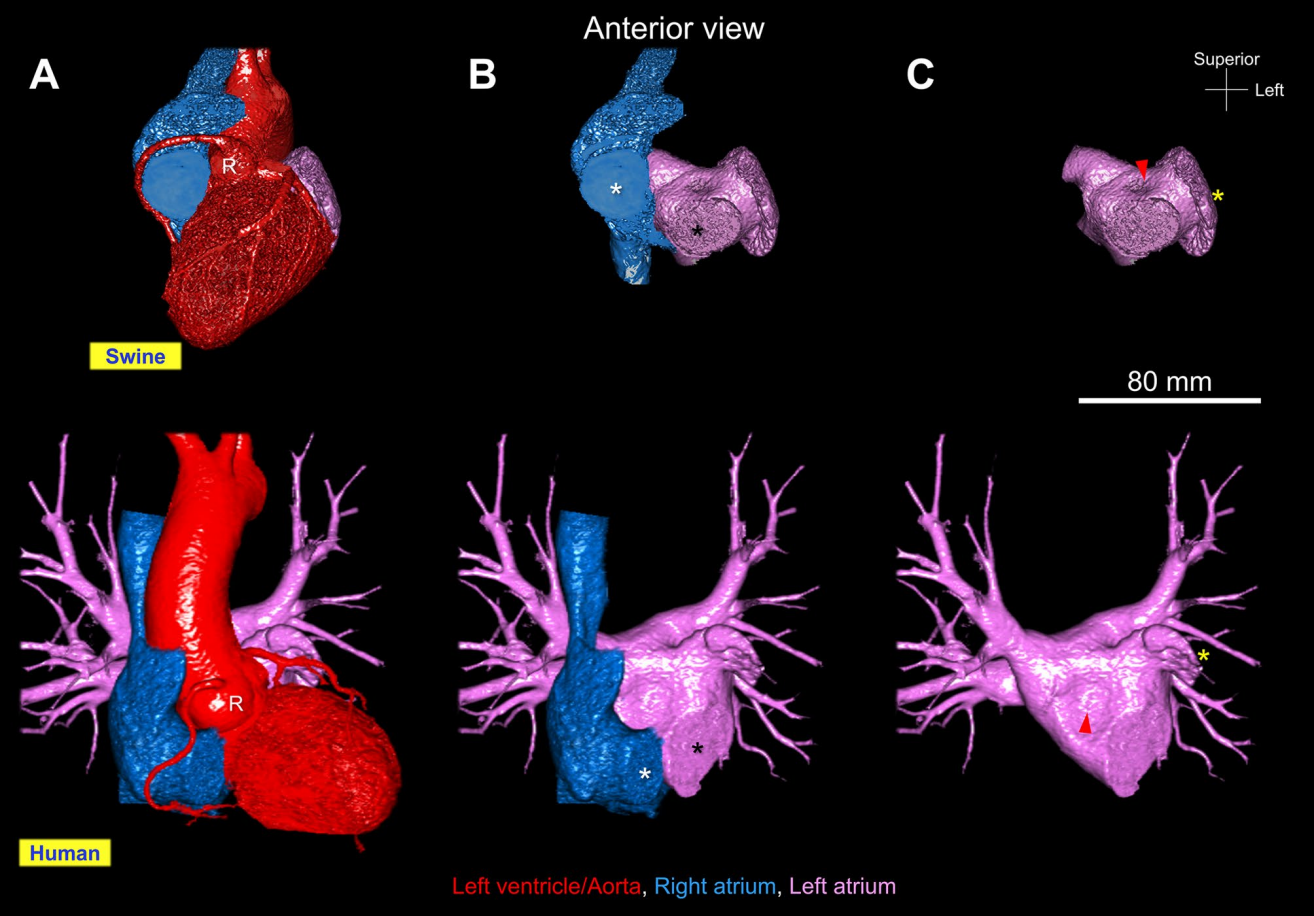
Comparative progressive virtual dissection of the swine and human hearts viewed from the frontal direction. Volume-rendered images of the swine (upper panels) and human (bottom panels) hearts viewed from the anterior direction (**A**-**C**) as shown in Fig. 2 are further virtually dissected by removing the right ventricle and pulmonary artery (A), after additional removal of the left ventricle and proximal aorta with coronary arteries (red) (B), and after further removal of the right atrium (blue), remaining the isolated left atrium (pink) (C). White and black asterisks mark the tricuspid and mitral valve annuli, respectively. Yellow asterisks denote the left atrial appendage. Red arrows indicate the imprint created by the aortic root. R, right coronary aortic sinus.

**Fig. 4:**
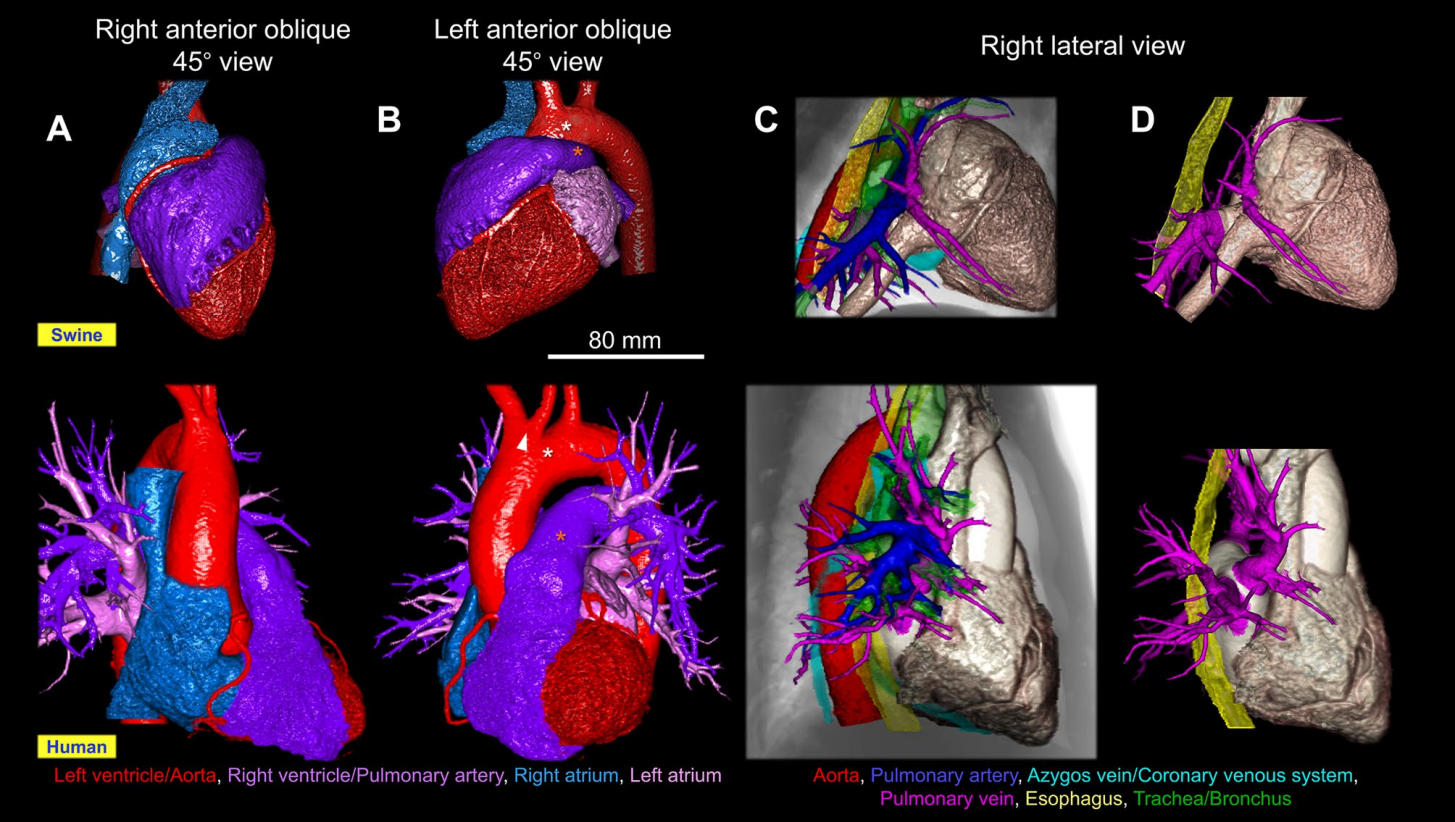
Comparison of the right and left anterior oblique views and right lateral views. Reflecting the difference in their physiological orientation and rotation within the thorax (Figs. 1, 2 and 3), the right (**A**) and left (**B**) anterior oblique views, as well as right lateral views (**C**, **D**) of the swine (upper panels) and human (bottom panels) hearts exhibit markedly different features. Note the differences in relationships between the aortic arch (white asterisks) and pulmonary trunk (red asterisks), distances between the esophagus (yellow) and the heart, and major branches from the aortic arch. In this patient, the aortic arch demonstrates a “bovine aortic arch” feature (white arrowhead) with a common trunk of the brachiocephalic and left common carotid arteries, which is neither identical to the major branches of the bovine aortic arch nor to those of the swine aortic arch (Fig. 5).

**Fig. 5:**
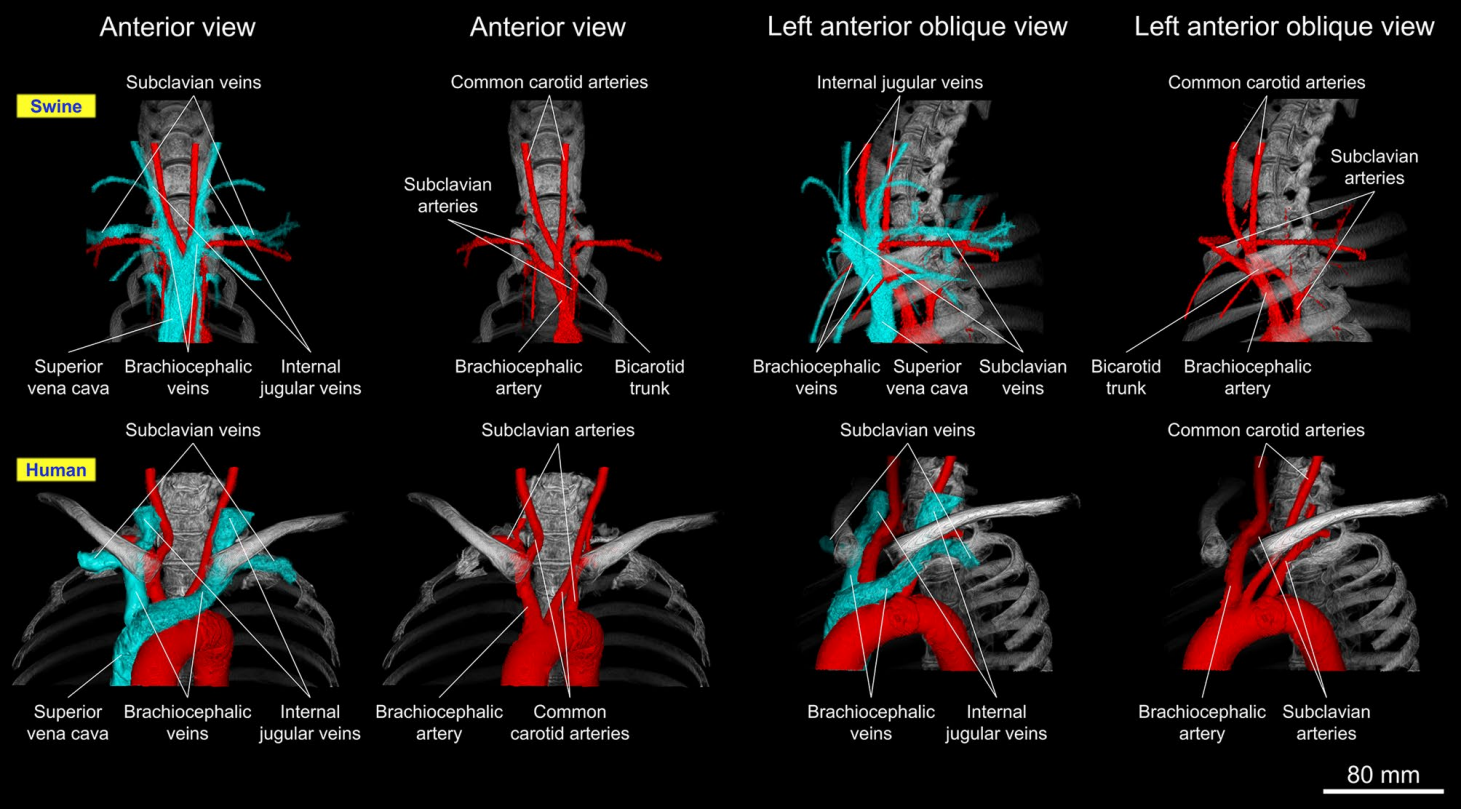
Comparison of the major branches of the aortic arch. The swine aortic arch (upper panels) has two major branches, involving the brachiocephalic and left subclavian arteries, with a characteristic bicarotid trunk originating from the brachiocephalic artery. The human aortic arch (bottom panels), on the other hand, typically has three major branches, involving the brachiocephalic, left common carotid, and left subclavian arteries. Note the swine lacks clavicles.

**Fig. 6:**
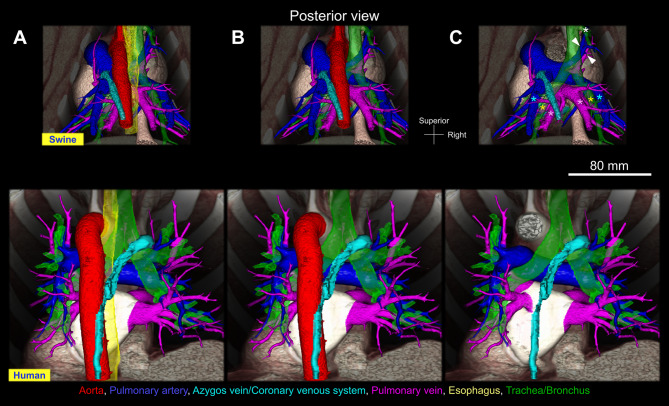
Comparative progressive virtual dissection of the swine and human hearts viewed from the posterior direction. Volume-rendered images of the swine (upper panels) and human (bottom panels) hearts viewed from the posterior direction (**A**-**C**) are demonstrated with progressive virtual dissection by removing the esophagus (yellow) (B) and the distal half of the thoracic aorta (red) (C) to visually denote characteristic differences in these colored structures. Blue, sky-blue, green, and pink structures indicate the pulmonary artery, left azygos vein, trachea and bronchi, and pulmonary veins, respectively. White asterisk denotes the characteristic tracheal bronchus (pig bronchus) of the swine, supplying exclusively the right upper lobe. Sky-blue, yellow, and pink asterisks indicate characteristic parallel relationships between the pulmonary arteries, bronchi, and inferior pulmonary veins, respectively. White arrowheads are the branches of the right pulmonary artery and right superior pulmonary vein associated with the right upper lobe.

**Fig. 7:**
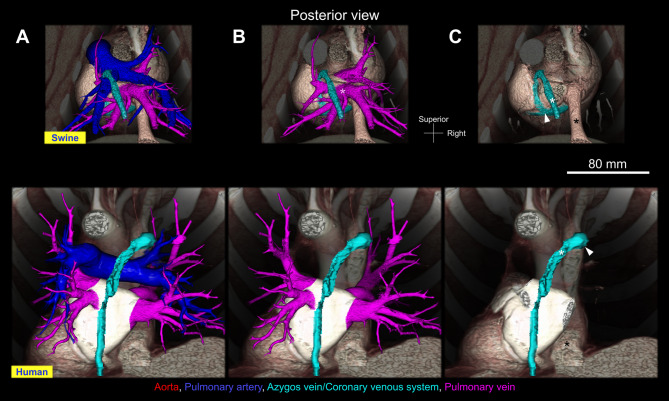
Comparative progressive virtual dissection of the swine and human hearts viewed from the posterior direction. Volume-rendered images of the swine (upper panels) and human (bottom panels) hearts viewed from the posterior direction (**A**-**C**) as shown in Fig. 6 are further virtually dissected by removing the trachea and bronchus (A), after additional removal of the pulmonary artery (blue) (B), and after further removal of the pulmonary veins (pink), leaving the isolated left azygos vein (sky-blue) (C). Pink asterisk marks the converging common orifice of the right and left inferior pulmonary veins. White arrowheads indicate the region where the left azygos vein (white asterisks) drains back to the coronary sinus in swine and to the superior vena cava in humans. Note the longer thoracic segment of the inferior vena cava (black asterisks) in the swine heart versus that in the human heart.

**Fig. 8:**
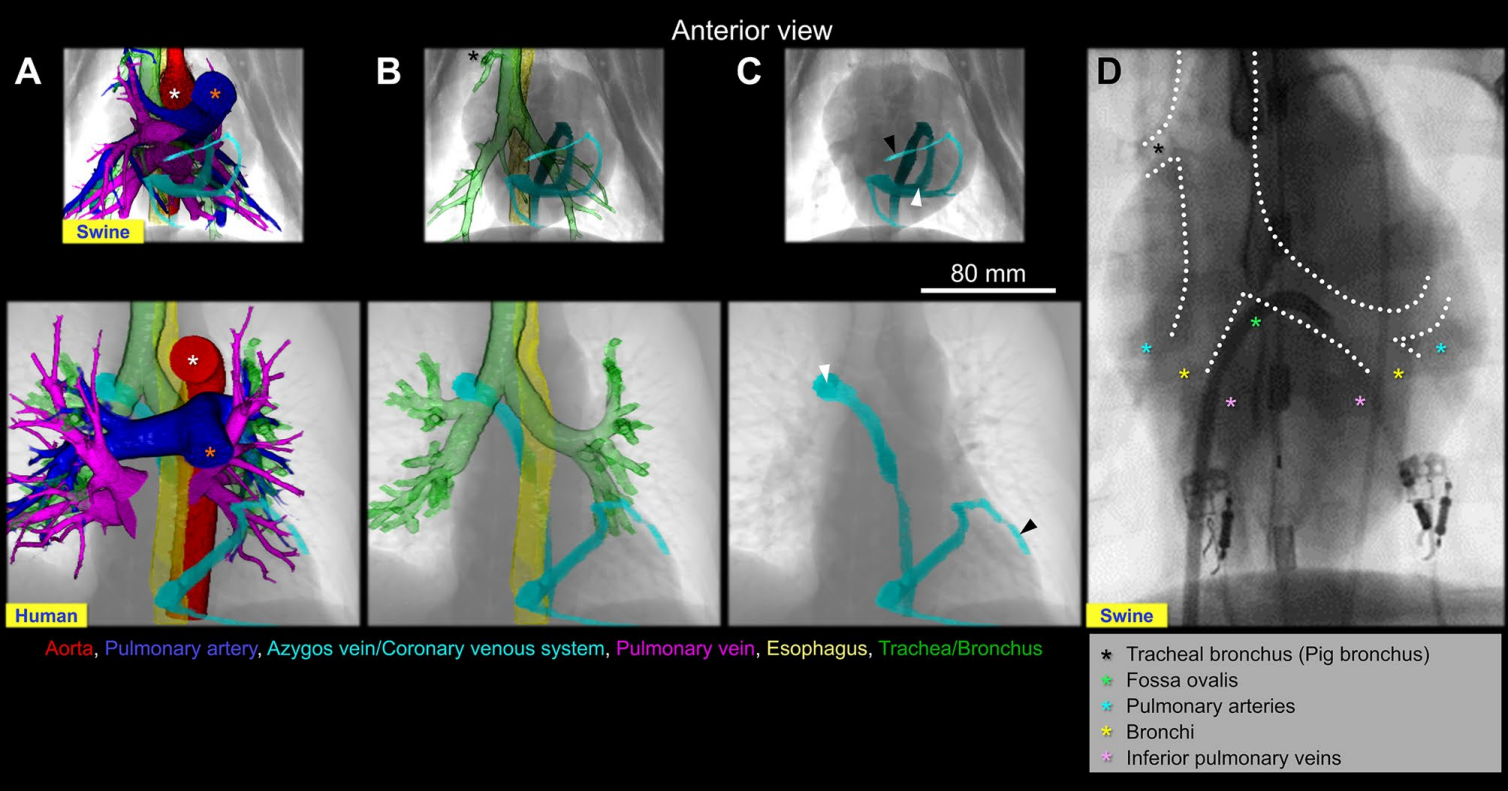
Comparison of the azygos vein drainage and swine bronchial anatomy. Volume-rendered images (A-C) of the swine (upper panels) and human (bottom panels) hearts viewed from the anterior direction (**A**-**C**) highlights the structures, including the pulmonary artery (blue), aortic arch and descending aorta (red), pulmonary veins (pink), trachea and bronchus (green), esophagus (yellow), and azygos vein and coronary veins (sky-blue). Virtual progressive dissection reveals the differences in the azygos vein drainage (white arrowheads) into the coronary sinus in swine and into the superior vena cava in humans. Black asterisks denote the tracheal bronchus (pig bronchus). Black arrowheads mark the anterior interventricular vein. The aortic arch (white asterisks) and pulmonary trunk (red asterisks) display the difference in their relationships between swine and humans (A). A fluoroscopic image (D) of the swine heart viewed from the anterior direction during an atrial transeptal puncture (yellow-green asterisk) can also reveal the characteristic parallel relationships between the pulmonary arteries (sky-blue asterisks), bronchi (yellow asterisks), and inferior pulmonary veins (pink asterisks) (Fig. 6).

**Fig. 9:**
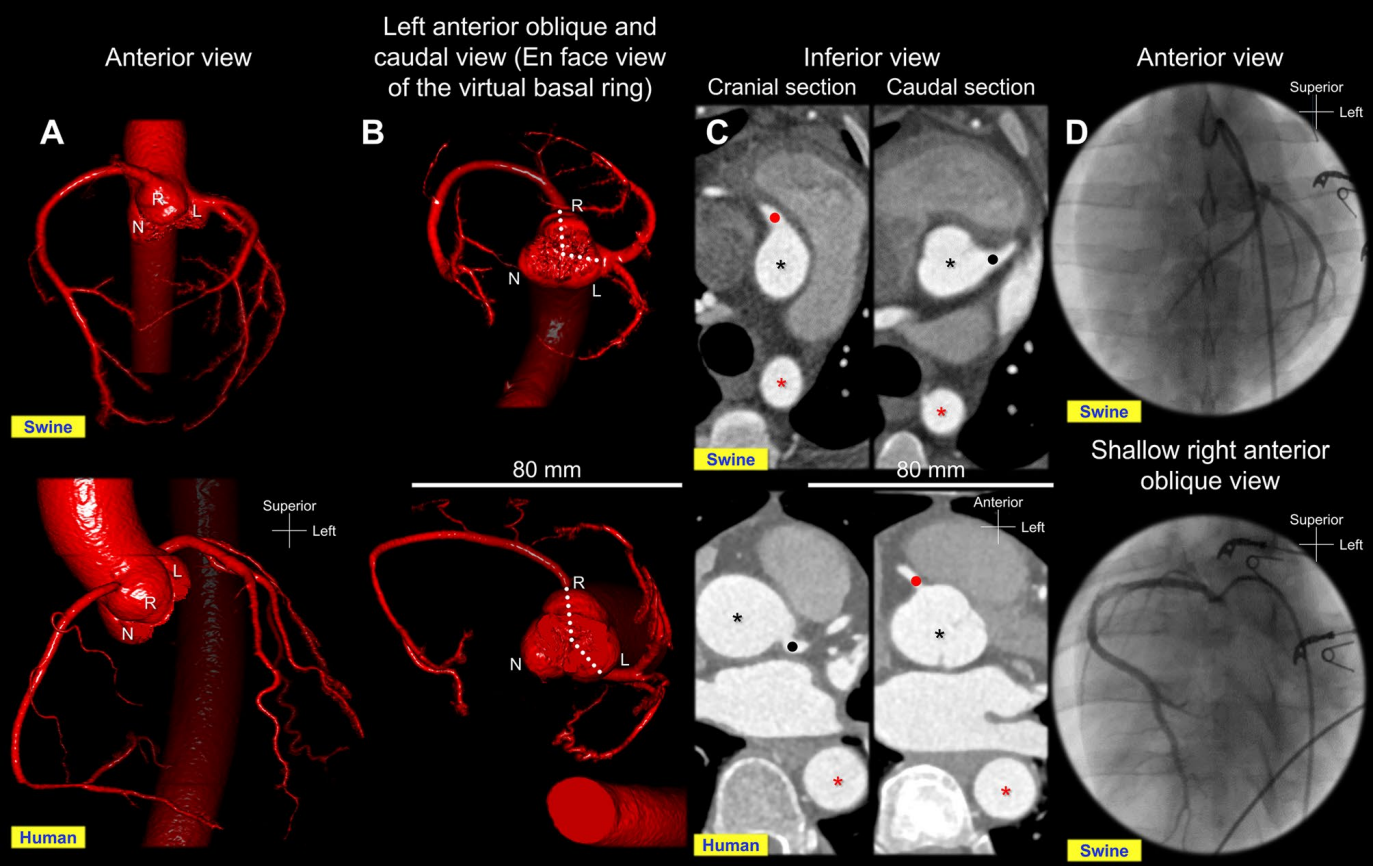
Comparison of the coronary arterial anatomy. Volume-rendered images viewed from the anterior (**A**) and left anterior caudal (**B**) directions and axial images (**C**) demonstrate the multiple differences between the swine (upper panels) and human (bottom panels) hearts. Red and black dots represent the orifices of the right and left coronary arteries, respectively, to reveal the difference in cranio-caudal orientation. Red and black asterisks mark the descending and ascending aorta, respectively, to reveal the difference in the plane involving them. Fluoroscopic images (**D**) obtained from the swine coronary angiography demonstrate the same feature as the volume-rendered image (A, upper panel). L, left coronary aortic sinus; N, non-coronary aortic leaflet; R, right coronary aortic leaflet.

**Fig. 10:**
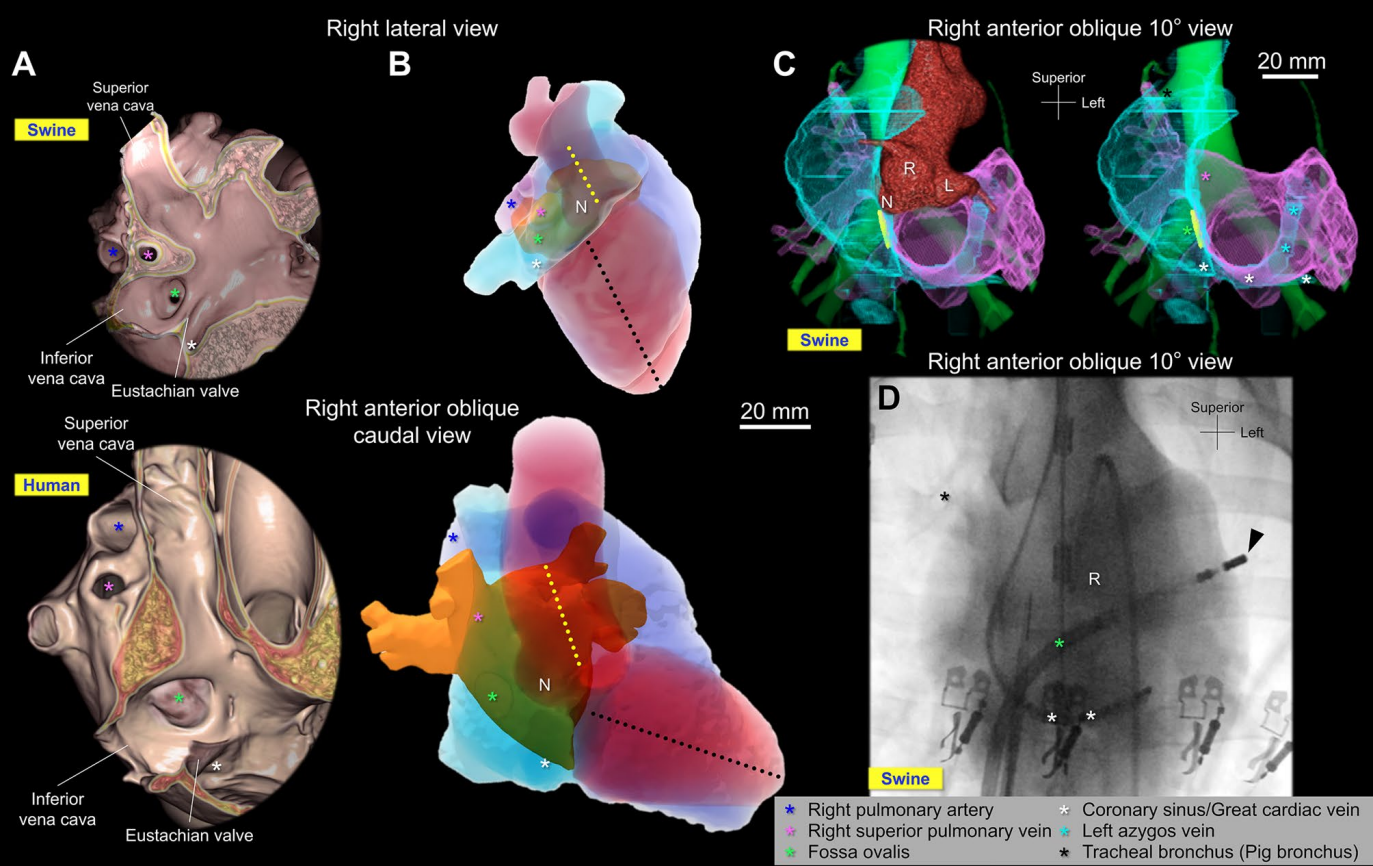
Comparison of the structural anatomy of the fossa ovalis. Volume-rendered images (**A**-**C**) of the swine (upper panels) and human (bottom panels) as well as a fluoroscopic image during swine atrial transseptal puncture (**D**) focus on the spatial relationships between the fossa ovalis (yellow-green asterisks) and surrounding structures (Fig. 8), including the right pulmonary artery (blue asterisks), right superior pulmonary vein (pink asterisks), coronary sinus (white asterisks), and non- (N) and right (R) coronary aortic sinuses. The coronary sinus catheter, pigtail catheter, intracardiac echocardiographic probe, and ablation catheter are placed in the coronary sinus, aortic root, right atrium, and left atrium, respectively. Yellow line and sky-blue asterisks in panel C mark the fossa ovalis and the left azygos vein, respectively. Note the fossa ovalis in the swine heart is situated between the superior structures (carina, aortic root, and right superior pulmonary vein) and the inferior structure (coronary sinus). A black asterisk indicates the tracheal bronchus (pig bronchus). Yellow and black dotted lines are the axes of the ascending aorta and left ventricle, respectively. The black arrowhead at the tip of the catheter seen outside the swine cardiac silhouette indicates the left superior pulmonary vein. L, left coronary aortic sinus.

**Fig. 11:**
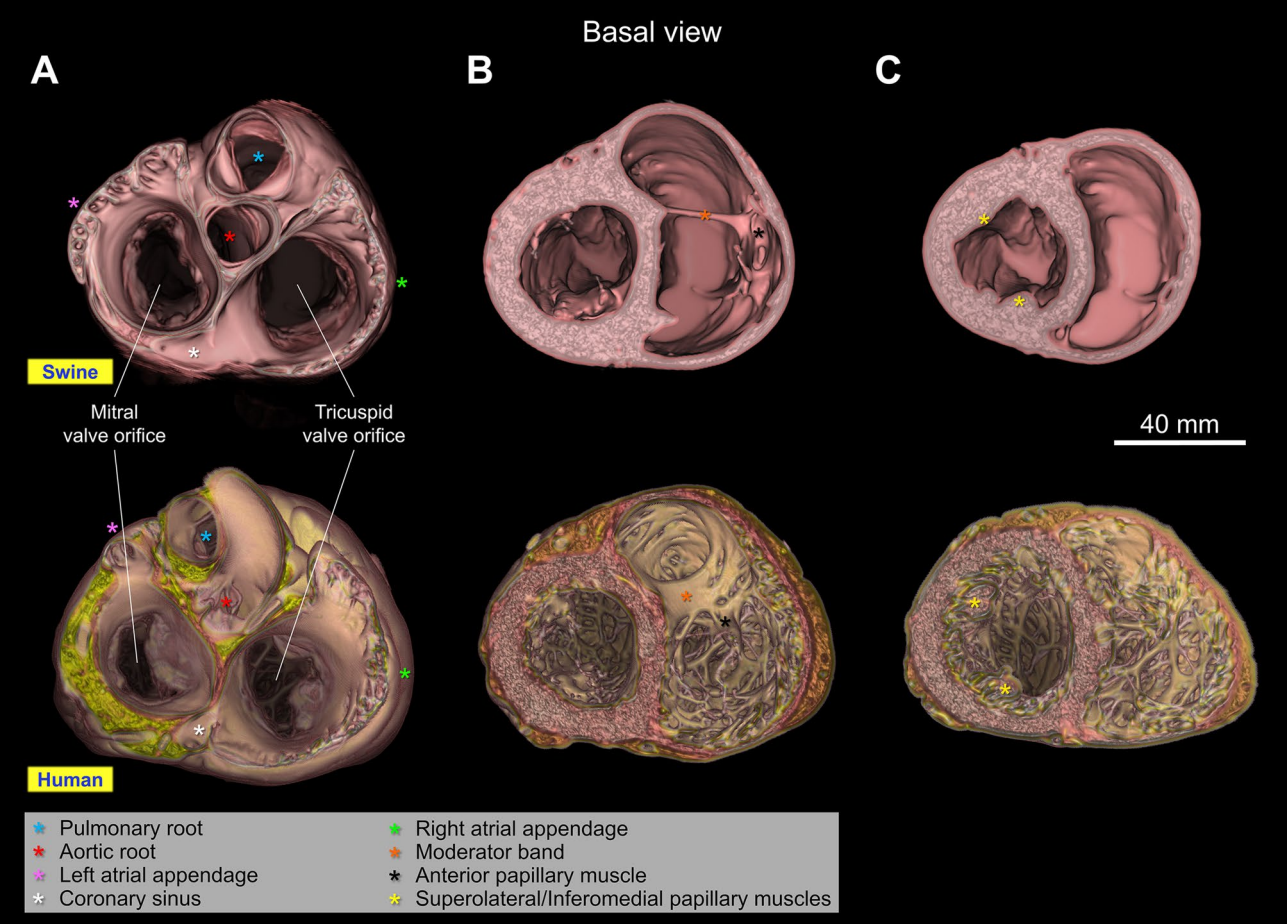
Comparison of the intracardiac structural anatomy with virtual dissection. Volume-rendered images of the swine (upper panels) and human (bottom panels) pressure-perfused and fixed hearts are sectioned into virtual planes parallel to the atrioventricular plane and viewed from the basal direction (**A**-**C**) to visually compare similarities and differences in features of the intracardiac structures. Sectional planes are set at the coronary sinus orifice level (A), chordae tendineae level (B), and left ventricular papillary muscle level (C). Sky-blue, red, yellow-green, pink, orange, white, and yellow asterisks indicate the pulmonary root, aortic root, right atrial appendage, left atrial appendage, moderator band, coronary sinus, and superolateral and inferomedial papillary muscles.

**Fig. 12:**
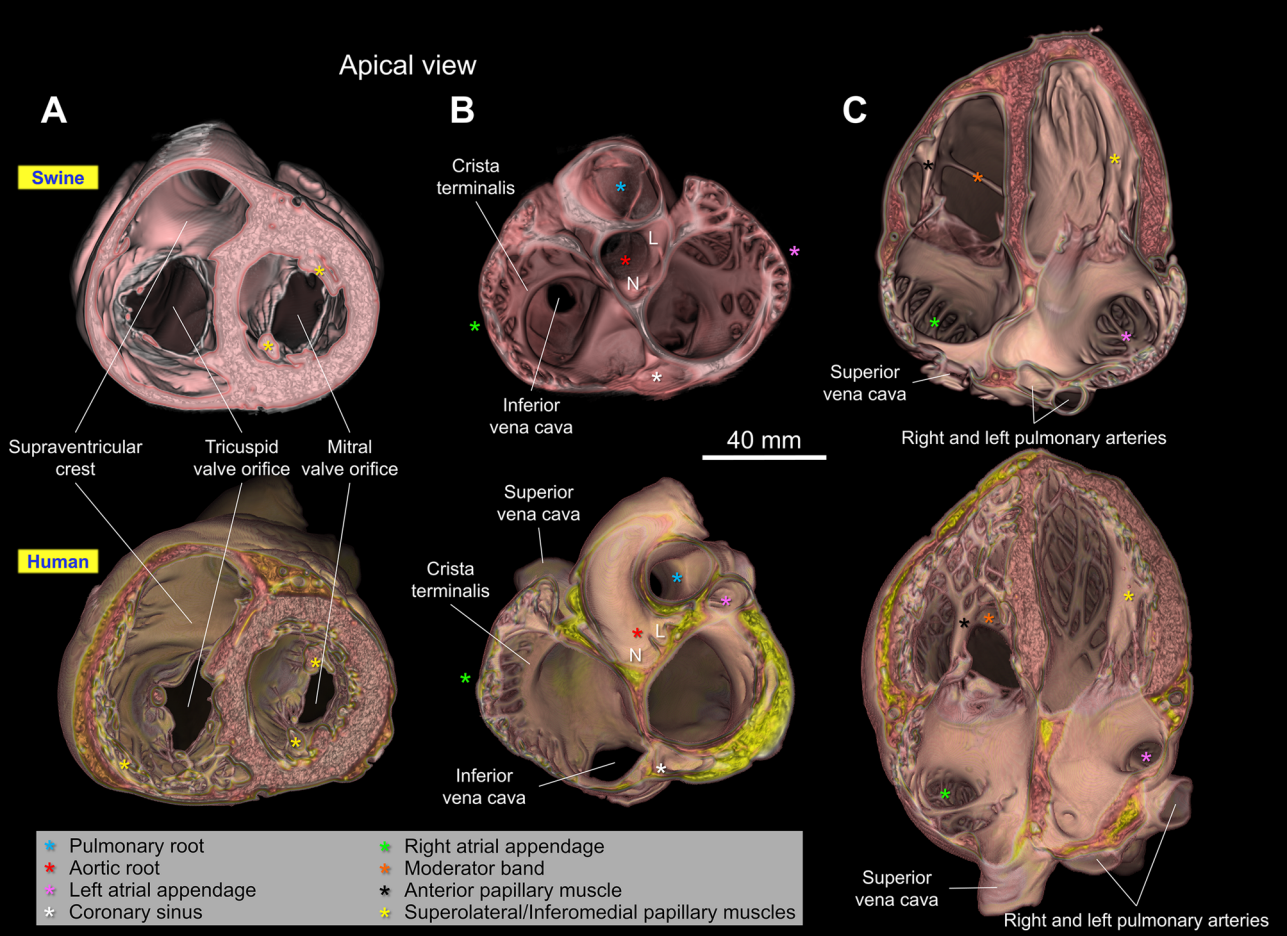
Comparison of the intracardiac structural anatomy with virtual dissection. Volume-rendered images of the swine (upper panels) and human (bottom panels) pressure-perfused and fixed hearts are sectioned into virtual planes parallel to the atrioventricular plane (**A**, **B**) and the four-chamber plane (**C**), and they were viewed from the apical (A, B) and inferior (C) directions, respectively to visually compare their similarities and differences in feature of the intracardiac structures. Sectional planes are set at atrioventricular valve level (A), coronary sinus orifice level (B), and central fibrous body level (C). Sky-blue, red, yellow-green, pink, orange, white, and yellow asterisks indicate the pulmonary root, aortic root, right atrial appendage, left atrial appendage, moderator band, coronary sinus, and superolateral and inferomedial papillary muscles. L, left coronary aortic sinus; N, non-coronary aortic sinus.

**Fig. 13:**
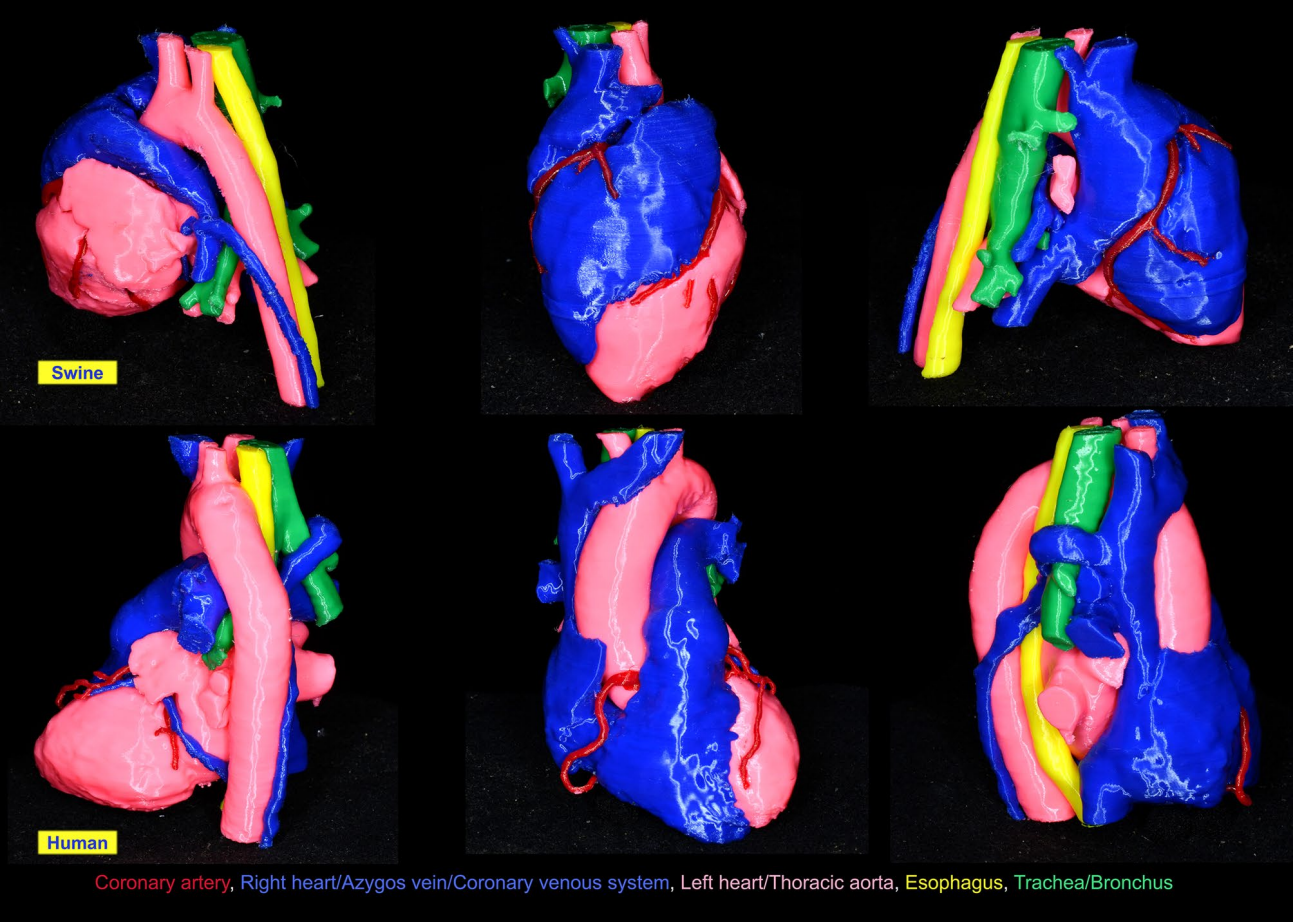
Resources to facilitate three-dimensional understanding. Three-dimensional relationships among the structures, including the left heart (pink), coronary artery (red), right heart (blue), trachea/bronchus (green), and esophagus (yellow) of swine (upper panels) and humans (bottom panels) are reconstructed from the cardiac computed tomographic datasets, exported as a digital stereolithography (STL) polygon model (Supplemental STL files 1 and 2), and printed (Supplemental video files 1 and 2) using commercially available software and printers (thermoplastic polyurethane) (Hanna et al., 2024). These models allow for immediate and intuitive understanding of the complex three-dimensional anatomy of the swine and human hearts and surrounding structures.

There was a trend towards increased use of animals in research facilities in the United States, mainly driven by greater use of small animals, such as mice^1^. However, traditional rodent models do not always produce relevant phenotypes for studying human conditions^2^. Therefore, large animals are used as the alternatives to nonrodent species in preclinical research^3^. In the setting of large animals, farm animals, such as swine, have gained favor over dogs, cats, or monkeys, due to moral consideration and their unique advantage of similarities in anatomical and physiological characteristics of organ systems^3–5^. Swine is one of the most utilized farm animals used in preclinical and translational studies because of its close size and longer lifespan, which are more comparable to those of humans^2^. Because the swine heart has a coronary circulation and blood flow similar to those of the human heart, it facilitates the use of medical devices in swine as efficient models^4,6,7^. Therefore, the swine model is critical for advancing discoveries from rodent models to large animals and finally to humans^8^. In this regard, comparative knowledge of swine cardiac anatomy with that of humans is fundamental when insights from translational cardiac research using swine need to be applied to human settings. Extensive knowledge has been accumulated on the normal anatomy of the swine heart, highlighting its similarities with the human heart, including its size and coronary arterial system^4,6,7,9–12^. However, comparisons based on recovered and distorted hearts have limitations for understanding the full range of swine cardiac anatomy in the living setting during the experiment. In fact, when swine and human hearts are compared three-dimensionally as they lie within each thorax without distortion, remarkable differences are recognized beyond the known similarities and differences (Table 1). Also, knowledge of animal anatomy is largely confined to veterinary literature and textbooks^5^, which are not always readily accessible to researchers working on translational studies. Thus, we present herein a comprehensive macroscopic living anatomy of the swine heart compared with the human heart, using virtual dissection, in which the heart is either left in situ in the thorax (Figs. 1, 2, 3, 4, 5, 6, 7, 8, 9, 10 and 13, Supplemental video files 1 and 2, Supplemental STL files 1 and 2) or removed without distortion (Figs. 10, 11 and 12).

**Table 1:** Comparison of intrathoracic anatomy between humans and swine.

		Cardiac apex directs left anteroinferiorly	Cardiac apex directs inferiorly
**Cardiac orientation and rotation**	**1-4, 13**	Cardiac apex lies in the left hemithorax	Cardiac apex lies along the median line
		Right ventricle is located posterior to the sternum	Both ventricles are located posterior to the sternum
		Right heart is positioned inferiorly to the left heart	Right heart is positioned superiorly to the left heart
**Thoracic cage**	**1**,** 2**	Thorax is compressed anteroposterioly	Thorax is compressed laterally
**Lung**	**1**,** 2**	Right and left lungs have 3 and 2 lobes, respectively	Right and left lungs have 4 and 2 lobes, respectively
**Trachea and bronchus**	**6-8**,** 10**,** 13**	Tracheal bronchus is rare	Tracheal bronchus is common (right upper lobe)
		Tracheal carina is wide	Tracheal carina is narrow
**Fluoroscopic anatomy**	**1-4**,** 8-10**	Porcine heart frontal view is identical to left anterior oblique and cranial view of human heart	Porcine heart frontal view is identical to left anterior oblique and cranial view of human heart
		Ventricular and atrial septal plane align in the left anterior oblique direction	Ventricular and atrial septal planes align in the sagittal direction
		Atrioventricular plane faces left anteroinferior direction	Atrioventricular plane faces anteroinferior direction
		Right anterior oblique view is effective to separate the atria from the ventricle	Right lateral view is effective to separate the atria from the ventricle
**Subclavian and carotid arteries**	**4**,** 5**,** 13**	3 (Right brachiocephalic, left common carotid, and left subclavian arteries)	2 (Brachiocephalic and left subclavian arteries)
		Bicarotid trunk is rare	Bicarotid trunk is common
**Thoracic aorta**	**1**,** 3**,** 8-10**,** 13**	Proximal aorta is more tortuous and tilted right	Proximal aorta is less tortuous and tilted, almost vertical
		Ascending aorta is longer and the aortic root is lower	Ascending aorta is shorter and the aortic root is higher
		Aortic root is located anterior to the left atrium	Aortic root is located superior to the left atrium
		Thoracic aortic plane faces left anterior oblique direction	Thoracic aortic plane faces left lateral direction
		Left coronary aortic sinus is located superior to the right coronary aortic sinus	Right coronary aortic sinus is located superior to the left coronary aortic sinus
**Esophagus**	**1**,** 4**,** 6**,** 13**	Esophagus courses right anterior to the descending aorta	Esophagus courses right to the descending aorta
		Esophagus is close to the left atrium	Esophagus is remote from the left atrium
**Pulmonary trunk**	**4**,** 11-13**	Pulmonary trunk is located inferior to the aortic arch	Pulmonary trunk is located at the same level of the aortic arch
		Pulmonary trunk ascends towards the superoposterior direction	Pulmonary trunk descends towards the inferoposterior direction
**Pulmonary veins**	**6-8**,** 13**	Size of 4 pulmonary veins are identical	Left superior pulmonary vein is small and inferior pulmonary veins have common orifice/trunk
		Orifices of 4 pulmonary veins are widely separated	Orifices of 4 pulmonary veins are close
		Pulmonary venous component is extensive	Pulmonary venous component is minimal
		Inferior pulmonary veins are not parallel to the bronchus	Inferior pulmonary veins are parallel to the bronchus
**Pulmonary arteries**	**6-8**,** 13**	Proximal pulmonary arteries are not parallel to the bronchus	Proximal pulmonary arteries are parallel to the bronchus
		Pulmonary bifurcation and carina are located superior ot the left atrium	Pulmonary bifurcation and carina are located posterior to the left atrium
		Pulmonary root is located left superior to the aortic root	Pulmonary root is located superior to the aortic root
**Left azygos vein (hemiazygos vein)**	**7**,** 8**,** 13**	Hemiazygos vein drains into the superior vena cava	Hemiazygos vein drains into the coronary sinus
**Coronary artery**	**9**,** 13**	Coronary artery generally exhibits right-dominant system	Coronary artery generally exhibits right-dominant system
		Coronary arteries are oriented in oblique fashion	Coronary arteries are oriented in vertical fashion
		Left coronary artery orifice is located superior to the right coronary artery orifice	Right coronary artery orifice is located superior to the left coronary artery orifice
**Left ventricular and ascending aortic axes**	**10**	Left ventricular and ascending aortic axes are more angulated	Left ventricular and ascending aortic axes are less angulated
**Fossa ovalis**	**10**	Fossa ovalis is located at a similar level to the non-coronary aortic sinus	Fossa ovalis is located inferior level to the non-coronary aortic sinus
**Atrial appendages**	**3**,** 11-13**	Right atrial appendage is more extensive than the left atrial appendage	Right and left atrial appendages are similarly extensive
**Ventricular trabeculations**	**11**,** 12**	Ventricular trabeculation is well developed and extensive	Ventricular trabeculation is less developed or coalesced
**Moderator band**	**11**,** 12**	Moderator band is thicker and originates from the mid-ventricular septum	Moderator band is thinner and originates from the basal ventricular septum
**Papillary muscles**	**11**,** 12**	Orientation of the left ventricular papillary muscles relative to the mitral valve is identical	Orientation of the left ventricular papillary muscles relative to the mitral valve is identical
**Superior and inferior vena cavae**	**4**,** 7**,** 10**,** 12**,** 13**	Thoracic segment of the inferior vena cava is shorter	Thoracic segment of the inferior vena cava is longer

## Methods

Computed tomographic datasets obtained from regular experiments using swine were reviewed and used for image analysis and anatomical comparison with human datasets. Swine experiments were performed in accordance with guidelines of both the University of California Institutional Animal Care and Use Committee and the National Institutes of Health Guide for the Care and Use of Laboratory Animals, and they were approved by the UCLA Chancellor’s Animal Research Committee (ARC#2022-019). The authors complied with the ARRIVE (Animal Research: Reporting of In Vivo Experiments) guidelines. Use of human datasets were approved from the University of California Los Angeles institutional review board (IRB#11-001354-AM-00004) in accordance with the principles outlined in the Declaration of Helsinki. Informed consent was obtained from all participants and/or their legal guardians.

In total, electrocardiogram-gated contrast-enhanced computed tomography datasets obtained from three live miniature breeds (Yucatán, age 9–26 months, body weight 34–61 kg) (Figs. 1, 2, 3, 4, 5, 6, 7, 8, 9, 10 and 13) in the supine position and plain computed tomography datasets obtained from two pressure-perfused and fixed hearts recovered from domestic farm breeds (Yorkshire, age 3–5 months, body weight 35–41 kg) (Figs. 10, 11 and 12) were used for image analysis^13^. For these Yorkshire pigs that were sacrificed to recover the hearts, they were medicated with intramuscular telazol (4–8 mg/kg) before intubation and ventilation. General anesthesia was maintained with inhaled isoflurane (0.8%-1.5%). They were euthanized by intravenous administration of a lethal dose of pentobarbital (100–200 mg/kg) and potassium chloride (75–150 mg/kg) under deep sedation with isoflurane (5%).

A commercially available 64-multidetector-row computed tomographic scanner (SOMATOM Definition AS, Siemens Healthineers, Erlangen, Germany) was used to obtain three-dimensional datasets that enabled non-invasive three-dimensional morphological evaluation. Image reconstruction, mainly using the volume-rendering method, was performed by using a commercially available workstation (Ziostation2 version 2.9.8.5; AMIN Co, Ltd; Ziosoft Inc., Tokyo, Japan), and images were compared with identical volume-rendered images reconstructed from the datasets obtained from the clinical cardiac computed tomography of patients (52-year-old female, 53-year-old male, 79-year-old female) with normal cardiac anatomy (Figs. 2, 3, 4, 5, 6, 7, 8 and 9) or from the computed tomography of a normal human heart (56-year-old female, 65-year-old female) rejected for transplantation following pressure-perfusion and fixation (Figs. 10, 11 and 12).

Comparability of the living anatomy of swine and human hearts (Figs. 1, 2, 3, 4, 5, 6, 7, 8, 9 and 10) was secured as follows. Electrocardiogram-gated cardiac computed tomography datasets were reconstructed from mid-diastolic datasets acquired at full inspiration in the supine position, with appropriate post hoc adjustment of potential rotation along the body axis. Comparability of the recovered hearts (Figs. 11 and 12) was ensured by applying identical pressure-perfusion and fixation methods to both hearts, achieving physiological diastolic morphology in all chambers without distortion. The hearts were virtually dissected in an identical fashion using common landmarks, such as the atrioventricular groove, coronary sinus, and cardiac apex. The sectioned planes were viewed from the same direction to obtain an en face image.

Although different terminology is often applied to several analogous structures in animals and humans, such as the cranial vena cava in animals and the superior vena cava in humans, in this manuscript, we consistently use human terminology by targeting researchers with a background and/or intention of human cardiac research and/or human clinical experience.

## Results

### Cardiac orientation and rotation

The swine heart stands on its apex (Figs. 1, 2 and 3), referred to as the typical Valentine position^10,14^. Thus, the left ventricular axis of the swine heart directs inferiorly, with additional minimum right and anterior angulation. This predominant inferior (right anteroinferior) axis of the left ventricle is highly different from the human left ventricle with the left anteroinferior axis. Thus, swine and human atrioventricular valve annuli face the predominant right anteroinferior and left anteroinferior directions, respectively (Fig. 3).

The swine cardiac apex lies along the median line, whereas the human heart is within the left hemithorax. This reflects the leftward (horizontal plane), ventral (sagittal plane), and cranial (frontal plane) rotations of the human heart, rendering the human right ventricle as the most anterior chamber behind the sternum with a superior shift of the cardiac apex^15^. Because of the lack of such a three-dimensional complicated rotation, both ventricles of the swine heart can be observed evenly from the anterior direction beneath the sternum (Figs. 1 and 2). Therefore, from the anterior direction, the human left ventricle is partially visible, covered by the right ventricle, while in swine, it is readily visible (Fig. 2)^16,17^. Essentially, the swine left ventricle lies anteriorly rather than posteriorly as in humans (Figs. 1 and 2). Also, based on differences in cardiac orientation and rotation, the right atrium and right ventricle in swine are positioned superiorly relative to their left-sided counterparts (Fig. 2)^4^. In contrast, in the human heart, it is the left atrium and left ventricle that are located slightly superior to their right-sided counterparts (Figs. 2, 3 and 4) ^16^.

### Thoracic cage and lung

Differences in the thorax between humans and swine result from the chest being dorsoventrally compressed in humans and laterally compressed in swine, reflecting a biped or quadruped stance in humans and swine, respectively^4^. This compression reflects the fact that the thorax is narrower in a swine and wider in a human in the horizontal direction (Fig. 1). When the thoracic cage is viewed from the anterior direction, the swine heart is seen on both sides of the sternum, highlighting its central, vertical location of the heart and the less extensive right lung. However, in humans, the heart is observed only on the left side of the sternum, largely covered by the bilateral lungs, except for the right ventricle and left ventricular apex, where there are general windows for transthoracic echocardiography (Fig. 2).

The swine lung is not identical to the human lung. In swine, the right lung has four lobes: upper (cranial/apical), middle, lower (caudal/diaphragmatic), and accessory lobes with incomplete interlobular fissures. The left lung has only the upper and lower lobes^3,5,9^. The upper lobe of the right lung has an exclusive bronchus originating from the trachea, referred to as the tracheal bronchus or pig bronchus^3,5,9^. This pig bronchus refers to a specific variation where the entire right upper lobe is supplied by a bronchus originating from the trachea (Figs. 6, 8 and 10). Interestingly, this feature could be observed in humans with a reported incidence of 0.2% ^18,19^. In accordance with this difference, the swine upper right lobe also has prominent branches from the right pulmonary artery and right superior pulmonary vein (Figs. 7 and 8).

The angulation of the tracheal bifurcation is much narrower in swine than in human (Figs. 6 and 8). The left pulmonary artery overrides the left main bronchus (hyparterial bronchus), and the right pulmonary artery runs anterior to the right main bronchus (eparterial bronchus) in humans^20^, whereas they show a characteristic parallel relationship in the swine heart (Figs. 6 and 8). Compared with humans, the pulmonary bifurcation and carina in swine are positioned more inferiorly and lie posterior to the left atrium, rather than superior to it (Figs. 6 and 8).

### Fluoroscopic anatomy

The swine heart orientation, especially the ventricular relationships, is similar to the human heart viewed from the left anterior oblique and cranial direction, which makes the human heart stand on its apex. The plane of the ventricular and atrial septa of the swine heart generally aligns in the sagittal direction, and the atrioventricular plane of the swine heart generally aligns on the horizontal plane, tilting towards the anteroinferior direction and facing the cardiac apex (Figs. 1, 2 and 3). The right and left anterior oblique views, commonly used during fluoroscopic procedures in both swine experiments and clinical practice, cannot provide identical features (Fig. 4). In the human heart, the right and left anterior oblique views are used to separate the atria from the ventricle, and the right heart from the left heart, respectively. This is due to the leftward rotation of the human heart on the horizontal plane. In the swine heart, however, the right lateral view is more effective in separating the atrium from the ventricle, and the anterior view or shallow right/left anterior oblique view is best for separating the right heart from the left heart (Figs. 2, 4, 8, 9 and 10). With this regard, the right anterior oblique view of the swine heart (Fig. 4) shares commonalities with the human frontal view (Fig. 2), both demonstrating partial coverage of the left ventricle by the right ventricle. Similarly, the left anterior oblique view of the swine heart (Fig. 4) is comparable to the left lateral view of the human heart.

### Subclavian and carotid arteries

While the human brachiocephalic, left common carotid, and left subclavian arteries emerge separately from the ascending aorta, the swine aorta possess only two branches (Figs. 4 and 5) ^21^. The left carotid artery is absent from the swine ascending aorta since the bicarotid trunk branches from the brachiocephalic artery (Fig. 5). The term “bovine aortic arch” is commonly used to describe the human aortic arch variant in which only two branches arise, primarily due to the left common carotid artery originating from the brachiocephalic artery (Fig. 4). However, this configuration differs from the true bovine and swine branching patterns, including the absence of a bicarotid trunk, and therefore represents a misnomer that should be avoided^22^. The branching pattern in cattle consists of a single brachiocephalic trunk that gives rise to the right and left subclavian arteries and a bicarotid trunk. Of note, despite utilizing the term “subclavian artery” in quadruped mammals, such as swine, dogs, horses, and cattle, these mammals lack the clavicle (Fig. 5).

### Thoracic aorta and pulmonary trunk

The thoracic aorta in swine is less tortuous, less tilted, and more vertically oriented compared to that of humans (Figs. 9 and 10). Consistent with the vertical orientation of the heart and less angulated relationship between the ascending aortic axis and left ventricular axis (Fig. 10), the swine aortic root is relatively higher within the thorax at the level of the left atrial roof and carina, rendering the ascending aorta shorter than the human ascending aorta (Figs. 3, 8, 9 and 10). Thus, the aortic imprint on the left atrium is located superiorly in swine, but it is located anteriorly in humans (Fig. 3). Because of the less tilted morphology of the thoracic aorta, the plane involving the thoracic aorta is nearly sagittal, facing the left lateral direction (Figs. 1 and 9) in the swine heart, compared to the human heart, where the plane is tilted and facing the left anterior oblique direction. Consistent with the nearly vertical direction of the ascending aortic axis with mild tilting to the posterior direction (Fig. 9), all the coronary aortic sinuses, hence the clean short axis image of the aortic valve, can be observed in a single axial plane in the swine heart in nearly identical size, whereas it is not feasible in the human heart because the tilting of the proximal aorta^23^, which is exaggerated with aging, towards the left anterosuperior direction (Figs. 1 and 9). In swine, the esophagus courses to the right of the descending aorta, whereas in humans, it is typically located right-anterior to the descending thoracic aorta. The descending aorta and esophagus are remote from the left atrium in the swine heart compared to their generally adjacent relationship in the human heart (Figs. 1 and 4).

The relationships between the aortic arch and pulmonary trunk, as well as the aortic and pulmonary roots, are also different. In swine, the aortic arch and pulmonary trunk are almost located at the same horizontal level, in contrast to the lower location of the pulmonary trunk relative to the aortic arch in humans (Figs. 4, 5, 6, 7 and 8). Because of this relatively higher location of the pulmonary trunk in the swine heart, the pulmonary trunk descends towards the caudal direction as it bifurcates to the pulmonary arteries (Figs. 6, 7 and 8). The human pulmonary trunk, in contrast, ascends towards the superoposterior direction (Figs. 2 and 8).

When creating the four-valve virtual dissection viewed from the atrial direction (Fig. 11) or ventricular direction (Fig. 12), the human pulmonary root is located more on the left lateral side relative to the aortic root or the plane of the atrial septum, with a more prominent three-dimensional crisscross angle between the arterial trunks, reflecting the tilting of the proximal aorta. In contrast, the swine pulmonary root lies on the aortic root along the plane of the atrial septum (Figs. 4, 11 and 12).

### Pulmonary veins and arteries

The swine heart has four pulmonary veins, the left and right superior and inferior pulmonary veins, comparable to the human hearts. The left superior is the smallest, followed by the middle-sized and isolated right superior pulmonary vein with an obvious myocardial sleeve, and the largest inferior pulmonary veins, which have a double-barreled orifice or a common orifice with a short common trunk (Fig. 7). Different from the human pulmonary veins, which has extensive area between them, also referred to as the dome/roof or posterior wall of the left atrium^24^, the pulmonary venous component in the left atrium of the swine heart is minimal. Thus, the four pulmonary veins have their orifices close to each other in swine, while the orifices are widely separated in humans. This may indicate the less extent of cannibalization of each pulmonary vein during development. The right superior pulmonary vein, as well as the right pulmonary artery, possess an exclusive branch related to the right upper lobe, which is supplied by the tracheal bronchus (Figs. 6 and 7). In the swine heart, the main branches of the pulmonary artery, bronchus, and pulmonary vein that are related to the bilateral lower lobes course in parallel fashion towards the inferolateral direction, with the pulmonary artery located most laterally, followed by the bronchus and the most medially located pulmonary vein (Figs. 6 and 8). This characteristic parallel relationship cannot be observed in humans. Instead, both proximal pulmonary arteries course in the horizontal plane, especially the right pulmonary artery, which lies horizontally on the left atrial roof in humans (Fig. 7).

### Left azygos vein (Hemiazygos vein)

The left azygos (hemiazygos) vein in swine drains to the coronary sinus, while in humans, it drains into the superior vena cava after overriding the right main bronchus (Figs. 7 and 8), which is a well-known difference^4,7,25,26^. Therefore, the left azygos vein demonstrates a characteristic candy-cane appearance, overriding the left pulmonary veins, left bronchus, and left pulmonary artery to converge with the coronary sinus (Figs. 7 and 8). Generally, this left azygos vein is thicker than the great cardiac vein, which continues to the lateral left atrioventricular groove.

### Coronary artery

Both swine and humans have right and left coronary arteries, and swine hearts typically exhibit a right-dominant system, which is also the case in 90% of human hearts (Fig. 9)^3,11^. Consistent with the orientation and rotation of the heart, however, the swine coronary arteries are vertical, and the human coronary arteries are oblique (Fig. 9). Thus, when viewed from the anterior direction, the right coronary orifice is located superior to the left coronary orifice in swine, but vice versa in humans. In a similar fashion, during cranio-caudal scans of axial images, the right coronary aortic sinus is first seen in swine, and in humans, it is the left coronary aortic sinus (Fig. 9).

Additionally, in accordance with the tortuosity of the thoracic aorta, the descending aorta is located posterior to the aortic root in swine, whereas it is situated left posterior to the aortic root in humans. When the aortic root is viewed from the virtual basal ring, the right and left coronary orifices can be seen to be at nearly a 90° angle, whereas in humans, it is greater than 140° (Fig. 9)^27,28^. These detailed differences should affect the findings observed during coronary angiography and catheter selection in the swine heart, given its short ascending aorta.

### Left ventricular and ascending aortic axes

The left ventricular and ascending aortic axis relationship is more or less parallel in the swine heart, compared to angulated relationships in the human heart with various extent^15,29^, when the swine and human hearts are viewed from the right lateral and right anterior oblique directions, respectively (Fig. 10). This is due to the cardiac orientation, rotation, the less-wedged short ascending aorta in the swine heart, and especially, the superior shift of the cardiac apex in the human heart^15,29,30^,

### Fossa ovalis

Based on the less wedged, highly located aortic root within the vertical heart, the relationships among the right pulmonary artery, right superior pulmonary vein, fossa ovalis, and coronary sinus to the non-coronary aortic sinus are different between swine and humans. Generally, in humans, the fossa ovalis is located at a similar level to the non-coronary aortic sinus^15^; the fossa ovalis of the swine heart is located lower than the non-coronary aortic sinus (Fig. 10). During the fluoroscopy viewed from the anterior or shallow right or left anterior oblique direction, the fossa ovalis of the swine heart is situated between the level of the carina and the coronary sinus orifice (Fig. 10).

### Atrial appendages

The extent of atrial appendages, as defined by the extent of pectinate muscles, is also different. While in humans, the right atrial appendage is extensive, continuing to the superior, lateral, and inferior right atrial vestibule, in contrast to the limited extent of the left atrial appendage, which is confined to the superolateral area of the left atrial vestibule (Figs. 11 and 12). The extent of the right atrial appendage in the swine heart is identical to that of humans. However, the extent of the left atrial appendage in the swine heart is far more extensive than in humans, continuing to the superior and lateral region of the left atrial vestibule (Figs. 3, 11 and 12). This may also reflect the limited extent of the pulmonary venous component.

### Ventricular trabeculations and papillary muscles

The human cardiac apex exhibits an extensive, well-developed trabeculation meshwork, which is fine and mural in the left ventricle and coarse and intra-chamber in the right ventricle. In contrast, the swine heart contains only coarse, poorly-developed, or coalesced trabeculations (Figs. 11 and 12)^4,31^.

The moderator band in the swine heart is typically much thinner and originates from the ventricular septum at the region closer to the tricuspid annulus^4^, whereas the human moderator band is thicker and originates from the mid-ventricular septum^32^.

Spatial orientations of the left ventricular superolateral and inferomedial papillary muscles relative to the ventricular septum and mitral valve, and right ventricular anterior papillary muscle, as well as the continuity of the moderator band, are identical in both species (Figs. 11 and 12).

### Superior and inferior vena cavae

The thoracic segment of the inferior vena cava above the diaphragm is longer in the swine heart than in the human heart (Fig. 7). Relationships between the superior and inferior vena cavae are more angulated in the swine heart (Figs. 4, 7, 10 and 12) than in the human heart^12^ when viewed from the right lateral and right anterior oblique directions, respectively. In the swine, the right superior pulmonary vein resides at the angle created by the confluence of both vena cavae. In contrast, the human right superior pulmonary vein is located relatively higher, posterior to the superior cavoatrial junction (Figs. 4, 7, 10 and 12).

Table 1 summarizes the present findings.

## Discussion

As demonstrated above, there numerous differences in terms of the anatomy of the swine and human hearts become apparent when evaluated without distortion. To the best of our knowledge, these details have never been examined in such a comprehensive fashion. The comparative observations shared here will contribute to more effective and relevant translational research using the swine heart.

### Translational implications

From a technical perspective, for example, echo windows for transthoracic echocardiography and the structures observed from these windows vary between humans and swine (Fig. 2), transesophageal echocardiography in the swine heart should be less informative due to the distance to the heart (Fig. 4), and intracardiac echocardiography needs to take into account the significant vertical axis of the heart. Fluoroscopic procedures, including coronary angiography, cannot be expected to exhibit identical features to those of humans when using the same fluoroscopic angulation as in the human situation, and vice versa (Figs. 4 and 9). The selection of catheters to access the coronary artery should consider the topographic difference in the origin and direction of each coronary artery, as well as the short ascending aorta and the plane of the thoracic aorta (Fig. 9). Atrial transseptal puncture of the swine heart, when performed with fluoroscopic guidance, should look remarkably high within the cardiac silhouette, and the importance of the carina and coronary sinus orifice should be appreciated in addition to the non-coronary aortic sinus (Fig. 8). The optimal angulation for each procedure should be different from that for the human heart. The coronary sinus catheter can readily jump into the left azygos vein in the swine heart rather than the great cardiac vein (Fig. 8).

From the anatomical perspective, the swine heart is unlikely to be an appropriate option for simulating pulmonary venous isolation or left atrial appendage closure, given its differences from the human heart. Similarly, intraventricular procedures, including leadless pacemaker implantation, should exhibit distinct procedural features considering the less extensive right ventricular apical trabeculation in swine. Future studies are necessary to validate these anatomical considerations in procedures using swine heart models. Overall, with improved understanding of cardiac anatomy of the swine heart, the implications for improving current experimental practice are numerous, commensurate with the large number of experimental studies.

### Three-dimensional digital approach for comparative anatomy

There is an increasing trend to utilize digital three-dimensional datasets, including the ones obtained from computed tomography, magnetic resonance imaging, and photogrammetry, for research and education not only in human but also in animal anatomy^33–35^. When using any methodology, only the quality of the three-dimensional datasets matters for maximizing their clinical/experimental relevance. The quality is not limited to spatial/temporal resolution, but also includes artifact/noise reduction, appropriate radiation exposure, optimized contrast volume and timing, and sophisticated three-dimensional visualization skills. Thus, for example, for cardiac imaging, multi-disciplinary approaches are often necessary to combine expertise in anatomy, radiology, and cardiology^16^. As demonstrated in the present datasets, a live animal study is the best way to analyze physiological morphology without losing spatial relationships with surrounding organs. When a recovered organ is analyzed, every effort should be made to preserve its physiological morphology without distortion. During reconstruction, it is important to define the target of visualization: surface contour, chamber geometry (endocast), or the tissue composing the organ. Images should be viewed from relevant, attitudinally appropriate directions rather than random directions. Based on these concepts, virtual dissection^36^ using comparable color scales as shown in the present images is a useful technique to enhance intuitive understanding in the context of comparable anatomical research.

### Limitations

The insights shared by this paper are not without limitations. First, we used limited datasets obtained from different breeds: the miniature breed Yucatan and the domestic farm breed Yorkshire. However, this difference is expected to affect only their growth rate (size), rather than their actual anatomical differences^3^. Given the limited sample size, providing quantitative datasets is beyond the scope of this visual and descriptive paper. Normative values for swine hearts across breeds and growth stages require further investigation. Second, age-related morphological changes involving the swine hearts cannot be assessed from current datasets obtained from relatively young swine. Therefore, the present data should be carefully interpreted, as the comparisons are between young swine and aged humans, and should not be directly applied to experiments involving elderly swine. Third, cardiac computed tomographic images are obtained from live Yucatan minipigs in the supine position. Thus, the physiological location of the heart when standing on all four feet could not be replicated. Obviously, the swine heart should be designed to bear the gravity from the dorsal to ventral direction, whereas the human heart is designed to accommodate the gravity from the cranial to caudal direction. However, as swine experiments are generally performed in the supine position, being familiar with the physiological supine anatomy of the supine heart should retain sufficient implication in the setting of translational research. Finally, we did not delve into microscopic differences between species, as they are beyond the scope and focus of this paper. Microscopic anatomy is known to be highly different between species, including differences in myocardial innervation, extent of Purkinje fibers, and atrioventricular conduction system^4,37–44^.

## Conclusions

A comprehensive comparative analysis of swine and human living heart datasets revealed significant differences in their three-dimensional structural anatomy. In addition to similarities, appreciation of these differences is fundamental for translational research using swine hearts. A similar comparative anatomical approach should be necessary for each specific species, considering the wide anatomical variation among species.

## Electronic Supplementary Material

Below is the link to the electronic supplementary material.

Supplementary Material 1

Supplementary Material 2

## Data Availability

All data presented or analyzed are available from the corresponding author.
